# Real-World Visual Experience Alters Baseline Brain Activity in the Resting State: A Longitudinal Study Using Expertise Model of Radiologists

**DOI:** 10.3389/fnins.2022.904623

**Published:** 2022-05-25

**Authors:** Jiaxi Su, Xiaoyan Zhang, Ziyuan Zhang, Hongmei Wang, Jia Wu, Guangming Shi, Chenwang Jin, Minghao Dong

**Affiliations:** ^1^Engineering Research Center of Molecular and Neuro Imaging of Ministry of Education, School of Life Sciences and Technology, Xidian University, Xi’an, China; ^2^Xi’an Key Laboratory of Intelligent Sensing and Regulation of Trans-Scale Life Information, School of Life Sciences and Technology, Xidian University, Xi’an, China; ^3^Department of Medical Imaging, First Affiliated Hospital of Medical College, Xi’an Jiaotong University, Xi’an, China; ^4^School of Foreign Languages, Northwestern Polytechnical University, Xi’an, China; ^5^Key Laboratory of Intelligent Perception and Image Understanding of Ministry of Education, School of Artificial Intelligence, Xidian University, Xi’an, China

**Keywords:** SVM, recursive feature elimination, visual expertise, resting state fMRI, ALFF

## Abstract

Visual experience modulates the intensity of evoked brain activity in response to training-related stimuli. Spontaneous fluctuations in the restful brain actively encode previous learning experience. However, few studies have considered how real-world visual experience alters the level of baseline brain activity in the resting state. This study aimed to investigate how short-term real-world visual experience modulates baseline neuronal activity in the resting state using the amplitude of low-frequency (<0.08 Hz) fluctuation (ALFF) and a visual expertise model of radiologists, who possess fine-level visual discrimination skill of homogeneous stimuli. In detail, a group of intern radiologists (*n* = 32) were recruited. The resting-state fMRI data and the behavioral data regarding their level of visual expertise in radiology and face recognition were collected before and after 1 month of training in the X-ray department in a local hospital. A machine learning analytical method, i.e., support vector machine, was used to identify subtle changes in the level of baseline brain activity. Our method led to a superb classification accuracy of 86.7% between conditions. The brain regions with highest discriminative power were the bilateral cingulate gyrus, the left superior frontal gyrus, the bilateral precentral gyrus, the bilateral superior parietal lobule, and the bilateral precuneus. To the best of our knowledge, this study is the first to investigate baseline neurodynamic alterations in response to real-world visual experience using longitudinal experimental design. These results suggest that real-world visual experience alters the resting-state brain representation in multidimensional neurobehavioral components, which are closely interrelated with high-order cognitive and low-order visual factors, i.e., attention control, working memory, memory, and visual processing. We propose that our findings are likely to help foster new insights into the neural mechanisms of visual expertise.

## Introduction

Visual experts refer to individuals with superior behavioral performance in visual recognition in specific domains (Curby and Gauthier, 2010). To become a visual expert, it requires visual learning with at least hundreds of cases of samples (Clark et al., 2012). A few real-world visual expertise models have been used to study the neural substrate underlying this behavioral expertise (Rossignoli-Palomeque et al., 2018). Increased level of activation was found in the left superior frontal gyrus and left cingulate cortex in radiologists, which is responsible for better working memory capability (Haller and Radue, 2005). Harel et al. (2010) demonstrated enhanced neuronal activity in the right precuneus of a group of car experts related to better memory retrieval strategies. Song et al. (2021) reported increased activation in the right anterior cingulate gyrus, but decreased activation in the superior parietal lobule in chess players (Song et al., 2020), which is closely related to improved visual processing and better attention control. These results derived from cross-sectional studies demonstrated that real-world visual experience alters the strength of evoked brain activity across widely distributed regions, which are supportive of high-order cognitive, such as attention control, working memory, and memory, and low-order visual components, such as visual processing (Khader et al., 2005; Cavanna and Trimble, 2006; Schipul and Just, 2016).

Low-frequency spontaneous fluctuations (0.01–0.1 Hz) of the restful brain play an important role in maintaining the ongoing internal brain representations (Oldfield, 1971; Tang et al., 2010; Evans et al., 2011), which are involved in the coding of previous experience and support learned skills (Dong et al., 2014). Particularly, patterns of spontaneous activity within the resting brain are shaped by experience-dependent changes in neural plasticity (Chakraborty, 2006). However, less attention has been paid to analyze how real-world visual experience alters the patterns of resting-state brain activity using longitudinal experimental design. In this regard, the baseline spontaneous neuronal activity reflects cortical excitability (Logothetis et al., 2001; Boly et al., 2007), and the level of cortical excitability may smear the spatial patterns of evoked brain activity (Di et al., 2013; Dong et al., 2015). We propose that the level of baseline brain activity is fundamentally important; therefore, this study aimed at investigating how short-term real-world visual experience modulates baseline neuronal activity in the resting state.

The amplitude of low-frequency fluctuations (ALFF) serves as an indicator of cortical excitability (Duff et al., 2008). Previous studies have used ALFF to measure the level of baseline brain activity in healthy subjects (Yang et al., 2007; Dong et al., 2015). In our study, a group of 32 radiology interns were recruited from our collaborative hospital. The resting-state MRI data were collected before and after 1 month of training in the X-ray department, and ALFF was calculated to quantify the level of baseline brain activity. To better capture the subtle changes in the strength of neuronal activity, a novel but sensitive machine learning analytical framework, support vector machine (SVM), was employed (Xu et al., 2019). We expected to see an altered level of activity in brain regions related to the multidimensional neurobehavioral component that supports visual recognition, such as attention control, working memory, memory extraction, and visual processing (Humphreys et al., 1999). To the best of our knowledge, this study is the first to investigate baseline neurodynamic alterations in response to short-term real-world visual experience using longitudinal experimental design.

## Materials and Methods

### Subjects

The subjects in this study consisted of a cohort group of radiology interns, who were medical students in the undergraduate program in national medical schools. They were recruited from the First Affiliated Hospital of Medical College, Xi’an Jiaotong University. Thirty-two radiology interns [15 male participants, age: 22.47 ± 1.02 years old, mean ± standard deviation (SD)]. The participants were aware of the purpose of the study and the reason why they were recruited. All the subjects are medical students in the undergraduate program in national medical schools, and they underwent a 4-week rotation in the First Affiliated Hospital of Medical College, Xi’an Jiaotong University. The subjects worked in the X-ray department during the rotation and reviewed approximately 35 cases per day, 6 days a week. Their daily training included interpreting X-ray images and drafting reports for each case. Senior radiologists were assigned to these interns as mentors and provided response to their clinical reports each day. The intern radiologists reviewed a minimum of 831 cases during the rotation period, as recorded in the Picture Archiving and Communication System (PACS). Moreover, all the subjects had no neurological or psychiatric brain diseases, had no history of head trauma, and had not taken recreational drugs or drugs that influence brain function during the time window of this study (Oldfield, 1971).

### Behavioral Tasks

This study employed a longitudinal experimental design, which is rare in visual expertise studies. Basically, the subjects underwent the behavioral assessment (including prescreening tasks and behavioral tasks) and MRI scanning before training, and behavioral assessment (only behavioral tasks) and MRI scanning after a 4-week visual training. Note that the purpose of prescreening and the behavior tasks were different. The prescreening procedures were conducted before MRI scanning, aiming to exclude confounding factors such as visual expertise from other known domains (e.g., cars, chess, birds, and mushrooms). Specifically, we used questionnaires to ensure that the subjects had no visual expertise of other known domains, such as aircrafts, animals, and plants. The behavioral tasks were conducted after MRI scanning, aiming to quantify the level of face expertise and radiological expertise, using the Cambridge face memory test (CFMT) (Duchaine and Nakayama, 2006) and radiological expertise task (Evans et al., 2011) respectively, as introduced in our previous studies (Wang et al., 2021; Zhang et al., 2022).

A standard behavioral task, i.e., radiological expertise task (Evans et al., 2011), was used to quantify the radiological expertise of the subjects before and after radiological training. The images selected for RET were identical for both tests. Basically, the subjects were shown 100 standard lung X-ray images and were asked to render a diagnostic decision (e.g., tumor present or absent) and prognosis (e.g., malignant vs. benign) for each image in RET. The 100 standard lung X-ray images were carefully chosen from the X-ray image library in the Medical Imaging Department of the First Affiliated Hospital of the College of Medicine under the guidance of three independent senior radiologists with more than 15 years of diagnostic radiology experience. These 100 images used for RET consisted of three ascending levels of difficulty with a portion of 50, 30, and 20%, respectively. Each lung X-ray image contained 0∼N nodules, and there was no mention of a diagnosis unrelated to pulmonary nodules. Sixty-five X-ray images containing only 1∼3 nodules were selected as positive cases, and 35 X-ray images without tumors were selected as negative cases. The pathologies in the images were carefully examined and reconfirmed by these three experts. The detailed procedure of CFMT and RET was introduced in our previous study (Zhang et al., 2022).

### MRI Data Acquisition

Before MRI scanning, all subjects underwent complete physical and neurological examination. Note that, to eliminate the potential influence of behavioral tasks on central representation, the behavioral task took place after MRI data acquisition. The MRI scanning was performed on the 3 Telsa MRI system (EXCITE, General Electric, Milwaukee, Wisc.) at the First Affiliated Hospital of Medical College, Xi’an Jiaotong University in Xi’an, China. To eliminate the time-of-day effect, the scanning was performed from 8:30 to 12:30 a.m. (Hasler et al., 2014). A resting scan and a structural scan were conducted. A standard birdcage head coil was used, along with restraining foam pads to minimize head motion and to diminish scanner noise. Prior to the scan, subjects were instructed to close their eyes, keep their heads still, and stay awake during the scanning process. After scanning, the subjects would be asked whether they had fallen asleep during the process.

For the resting MRI scanning, the following parameters were used. Each volume contains 35 axial slices, scan duration = 370 s, repetition time (TR)/echo time (TE) = 2,000 ms/30 ms, field of view = 240 mm, total brain volume collection = 185, matrix = 64 × 64, flip angle = 90°, voxel size = 3.8 × 3.8 × 5.0 mm^3^, gap = 0 mm, thickness = 4 mm, layer spacing = 0 mm. The resting-state fMRI scans lasted for 8 min and 20 s. High-resolution T1-weighted structural imaging data used 3D magnetization preparation to quickly acquire gradient echo sequence for acquisition.

### MRI Data Preprocessing

Statistical Parametric Mapping (SPM12)^1^ and the Data Processing Assistant for Resting-State fMRI (DPARSF 4.5)^2^ were used for MRI data preprocessing. The first 10 images were deleted to eliminate non-equilibrium effects of magnetization and allow the participants to adapt to the experimental environment. The images were corrected for the acquisition delay between slices, motion corrected and co-registered to the subject’s anatomical images in native space. Two subjects had head motion exceeding the threshold of 0.2 mm (frame-wise displacement, i.e., Power FD). For the remaining 30 subjects, a two-sample *t*-test was used to verify that there was no significant difference in head movement between the two groups for the remaining subjects. Next, all the functional images were normalized to the MRI space using the deformation field maps obtained from structural image segmentation, following the segmentation routine in SPM12. The normalized images were resampled to 3 mm isotropic voxels, which were then spatially smoothed with a 6-mm full width-at-half-maximum Gaussian kernel. Finally, the linear trend was removed (Dale et al., 2000), and temporal filtering (0.01–0.08 Hz) was performed on the time series of each voxel to reduce the effect of low-frequency drifts and high-frequency noise (Zou et al., 2008).

### Feature Extraction

#### Generation of Voxel-Wise Amplitude of Low-Frequency Fluctuations Map

Resting-State fMRI Data Analysis Toolkit (REST)^3^ was used to compute ALFF (Song et al., 2011). ALFF measures the level of intrinsic or spontaneous neuronal activity in a given voxel (Jiang et al., 2004). The ALFF serves as an indicator of cortical excitability (Duff et al., 2008), and the volume of regional cerebral blood flow is correlated with ALFF in the brain region from the resting-state data (Li et al., 2012); therefore, it is taken as the index for the level of baseline brain activity. To calculate ALFF, after preprocessing, a fast Fourier transform (FFT) was used to transform from time domain to frequency domain for a given voxel, and the specific parameters are as follows: the taper percentage was zero, and the FFT length was set to short. Then, the square root of the power spectrum at each frequency was calculated, and the average value was taken in the range of 0.01–0.08 Hz. The average square root of a given voxel was taken as ALFF (Jia et al., 2020). To minimize the impact of variability among participants and reduce noise interference, we divided the ALFF of a given voxel by the average ALFF value of whole brain voxels to obtain the standardized value.

#### Generation of Region-Wise Amplitude of Low-Frequency Fluctuations Map

The voxel-wise ALFF map was averaged into a region-wise ALFF map. The Brainnetome atlas was used to divide the ALFF map into 246 regions of interest (ROIs) (Fan et al., 2016), and the average ALFF value of each region was obtained by averaging the ALFF value in this region (Li et al., 2012). Mean ALFF values from the 246 ROIs then served as the input vector to the classification procedure.

#### Feature Selection

**FIGURE 1:**
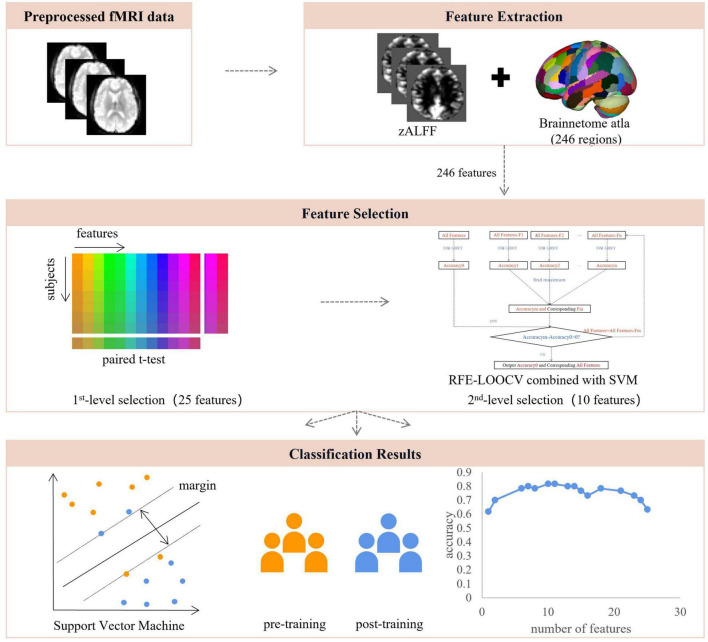
The pipeline of data analysis. After the resting-state fMRI data were preprocessed, voxel-wise and region-wise amplitudes of low-frequency fluctuations were extracted and used for feature selection, which consisted of two steps, including region-wise paired *t*-test and recursive feature elimination embedded in a leave-one-out cross-validation framework, resulting in 10 features of highest discriminative power. These features were used for SVM modeling with LOOCV. ALFF, amplitude of low-frequency fluctuations; RFE, recursive feature elimination; LOOCV, leave-one-out cross-validation.

Feature selection is necessary in MRI data analysis to avoid dimension disaster (Mladenić, 2006), reduce training time, and increase classification performance (Jiang et al., 2004; Dosenbach et al., 2010). A two-stage feature selection procedure was conducted, identifying features with the highest discriminative power. For the first-level selection, the paired sample *t*-test was performed between the region-wise post- and pre-training ALFF maps in a leave-one-out fashion. The combined region-wise features that survived the statistical threshold (*p* < 0.05) from each iteration were used as the input for a second-level feature elimination. Note that the remaining ALFF was regressed against the outcome of CFMT individually to remove the confounding effect from other domains of visual expertise, i.e., face in this study. Second, a recursive feature elimination-support vector machine (RFE-SVM) approach was used. This process recursively eliminates the least useful features until further elimination reduces the accuracy (Ding et al., 2015). The specific steps were as follows:

1. The training set was regressed against the outcome of CFMT.
2. The resulting beta-maps were normalized across all brain feature data between 0 and 1 through normalization of mean variance.
3. RFE reduced the dimension of features again and used the classifier itself to discard irrelevant features (Figure 1). Our implementation of RFE is described by the following pseudo-code:

a. Input all training samples and class labels, train SVM classifier, calculate the classification accuracy of the model accuracy_0_;
b. Sequentially subtract one feature, inputting the other into LOOCV-SVM, calculating the classification accuracy_i_ of the model, finding all accuracy_i_ greater than or equal to accuracy_0_, and determining the corresponding removed feature feature_i_;
c. Delete these features and update the feature set; and
d. Repeat the above steps until further elimination reduces the accuracy.

As a result, we were able to identify a set of brain regions of the highest discriminative power.

#### Support Vector Machine

Basically, SVM is a binary classification model (Cortes and Vapnik, 1995). The basic idea is to find the separation hyperplane with the largest interval in the feature space to make the data binary classification efficiently (Li et al., 2007). Linear SVM is often used in neuroimaging data in that it produces interpretable results (Rasmussen et al., 2011). Therefore, this study adopted the linear SVM classifier model of soft interval separation and hinge loss function. LIBSVM toolbox^4^ was used in this study (Chang and Lin, 2011).

The leave-one-out cross-validation (LOOCV) was used to assess the performance of the classifier (Dai et al., 2012). In LOOCV, each sample was designated as the test sample, while the remaining samples were used to train the multi-classifier. To quantify the performance of the classifier, according to the prediction results of LOOCV, the accuracy, sensitivity, and specificity were defined as follows:


(1) $$A\,c\,c\,u\,r\,a\,c\,y=\frac{\mathrm{TP}+\mathrm{TN}}{\mathrm{TP}+\mathrm{FN}+\mathrm{TN}+\mathrm{FP}}$$

(2) $$S\,e\,n\,s\,i\,t\,i\,v\,i\,t\,y=\frac{\mathrm{TP}}{\mathrm{TP}+\mathrm{FN}}$$

(3) $$S\,p\,e\,c\,i\,f\,i\,c\,i\,t\,y=\frac{\mathrm{TN}}{\mathrm{TN}+\mathrm{FP}}$$

where TP, FN, TN, and FP denoted the number of samples correctly predicted, the number of trained subjects classified as untrained ones, the number of untrained subjects correctly predicted, and the number of untrained subjects classified as trained ones, respectively. In this study, the area under the curve (AUC) was also used to represent the classification ability of SVM. A greater AUC value also represented a better classification ability.

### Statistical Analysis

The non-parametric permutation test (Filgueiras et al., 2014) was used to evaluate the statistical significance of the classification results. The features with the highest discriminative power were used in this step, i.e., the 10 features after feature selection. Each subject was treated as an independent sample. For a given sample, the label was randomly set to 1/-1 (1: post-training data, -1: pre-training data), while the label of the testing sample remained unchanged to determine the outcome of SVM. The procedure was repeated 1,000 times. Accordingly, the statistical significance of the original accuracy was calculated as the probability that the SVM classification result was greater than or equal to the original accuracy in the 1,000 replacement. The average accuracy was obtained in all permutations, and the *p*-value was calculated as a proportion larger than the average accuracy obtained by our method. The threshold of *p* < 0.05 was used to determine the significance.

### Regression Analysis

To assess the relationship between behavioral measurement and brain activity, Pearson’s correlation analysis was conducted between alterations in outcomes of CFMT and RET and alterations in region-wise ALFF. The significance level was set at *p* < 0.05 after multiple comparison correction (false discovery rate, FDR).

## Results

### Results of Behavioral Tasks

**FIGURE 2:**
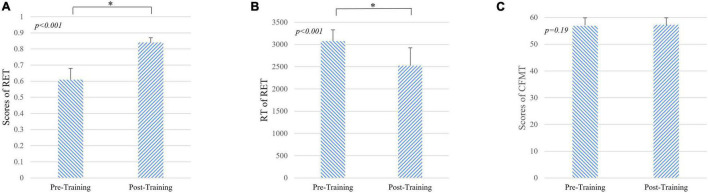
Results of behavioral tasks pre- and post-training. **(A)** The level of radiological expertise assessed by the radiological expertise task. The radiology interns had a significantly greater scores after training compared with scores before training (*p* < 0.001, Mann-Whitney *U*-test), indicating improved performance in visual recognition of radiological images. **(B)** Response time of radiological expertise task pre- and post-training. The radiology interns had a significantly faster in behavioral response after training compared with that before training (*p* < 0.001, Mann-Whitney *U*-test). **(C)** The level of face expertise measured by the Cambridge face memory test. No significant differences were found (*p* = 0.19, Mann-Whitney *U*-test). RET, radiological expertise task; RT, response time; CMFT, Cambridge face memory test. Error bars indicate the standard deviation. * indicats the significant differences between groups (*p* < 0.001).

During 1 month of training, the subjects reviewed at least 831 cases (926 ± 73, mean ± *SD*). As shown in Table 1, after 1 month of training, the performance of the radiologist interns significantly improved as revealed by higher scores in RET (*p* < 0.001, Mann-Whitney *U*-test) and shorter response time in RET (*p* < 0.001, Mann-Whitney *U*-test). Whereas the level of face expertise remained the same after 1 month of training in the domain of radiology (*p* = 0.19, Mann-Whitney *U*-test) (Figure 2).

**TABLE 1:** The results of behavioral tasks within the subjects pre- and post-training.

	Pre-training (*n* = 30)		Post-training (*n* = 30)		*p*-values
	Mean	*SD*	Mean	*SD*	
Cases reviewed	N/A	N/A	926	73	–
RET	0.61	0.05	0.84	0.04	<0.001
*RT of RET(s)	3.08	0.30	2.53	0.34	<0.001
CFMT	56.90	4.29	57.30	4.67	0.19

Note that the Mann-Whitney U-test was used to investigate group difference between groups. *Denotes the items showing significant difference between groups after Mann-Whitney U-test (p < 0.001). SD, standard deviation; s, seconds; RET, radiological expertise task; RT, response time; CFMT, Cambridge face memory test.

### Performance of Support Vector Machine

**FIGURE 3:**
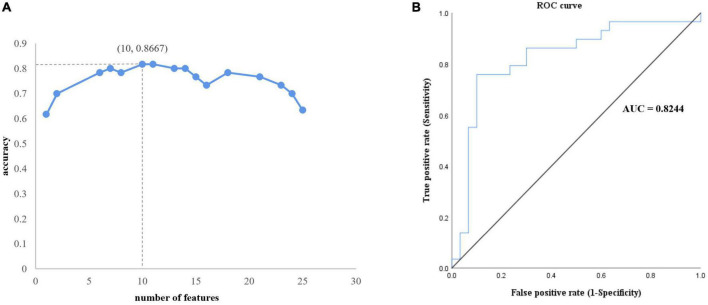
Performance of the proposed analytical framework. **(A)** Ten features corresponding to best classification accuracy. **(B)** The receiver operating characteristic curve. The area under the curve is 0.8244, which indicates outstanding performance.

After feature selection, 10 features remained corresponding to the highest accuracy (Figure 1). The classification accuracy of SVM after LOOCV reached 86.7% (Figure 3A), and the AUC was 0.8244 (Figure 3B). The specificity and sensitivity of SVM after LOOCV were 80.00 and 83.33%, respectively. The classification results were tested 1,000 times, and no repetition reached the classification accuracy of 86.7%. Thus, the statistical significance was *p* < 0.001, indicating that the results of our study were significantly higher than the chance value.

**FIGURE 4:**
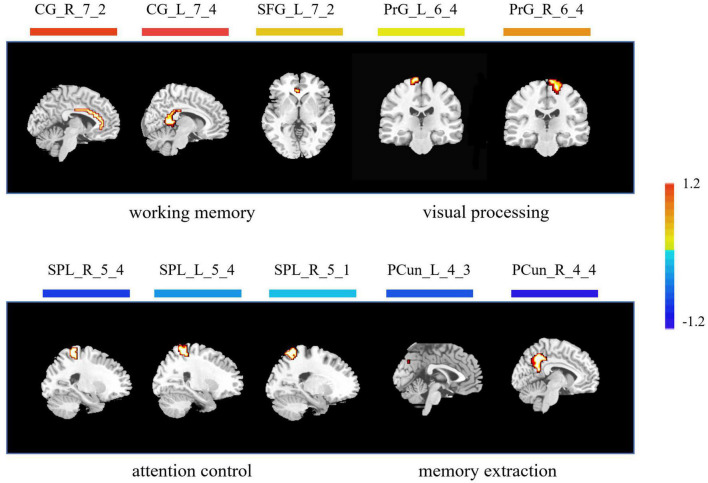
Brain regions with highest discriminative power pre- and post-training. The color bar indicates the weight of the feature. Note that positive weights refer to higher level of ALFF after training, and negative weights refer to lower level of ALFF after training. CG, cingulate cortex; SFG, superior frontal gyrus; PrG, precentral gyrus; SPL, superior parietal lobule; PCun, precuneus; L, left; R, right.

As for the brain regions, 10 regions were identified with the highest discriminative power, including the left cingulate cortex (CG_L_7_4), the right cingulate cortex (CG_R_7_2), the left superior frontal gyrus (SFG_L_7_2), the right precentral gyrus (PrG_R_6_4), the left precentral gyrus (PrG_L_6_4), the right superior parietal lobule (SPL_R_5_4), the right superior parietal lobule (SPL_R_5_1), the left superior parietal lobule (SPL_L_5_4), the right precuneus (PCun_R_4_4), and the left precuneus (PCun_L_4_3) (Figure 4 and Table 2).

**TABLE 2:** Brain regions that show highest discriminative power pre- and post*-*training.

Cognitive component	Labels	Brain region	Brodmann area	Hemisphere	Weight
Working memory	CG_L_7_4	Posterior cingulate cortex	BA23	L	0.59
	CG_R_7_2	Anterior cingulate cortex	BA24	R	0.99
	SFG_L_7_2	Superior frontal gyrus	BA8	L	0.26
Memory	PCun_L_4_3	Precuneus	-	L	-0.85
	PCun_R_4_4	Precuneus	BA31	R	-1.02
Attention control	SPL_L_5_4	Postcentral area	BA7	L	-0.55
	SPL_R_5_4	Superior parietal lobule	BA7	R	-0.92
	SPL_R_5_1	Postcentral area	BA7	R	-0.54
Visual processing	PrG_L_6_4	Precentral gyrus	BA4	L	0.12
	PrG_R_6_4	Precentral gyrus	BA4	R	0.44

*Note that positive weights refer to higher level of ALFF after training, and negative weights refer to lower level of ALFF after training. L, left; R, right.*

### Results of Regression Analysis

No significant correlations were found between alterations in the outcomes of CFMT and RET and alterations in region-wise ALFF after multiple comparisons.

## Discussion

The acquisition of visual expertise requires at least hundreds of cases of training within a specific domain (Annis and Palmeri, 2018). In real-world visual learning, several behavioral components, including high-order cognitive, such as memory (Viggiano et al., 2006), attention (Rose et al., 2004), and working memory (Ennaceur, 2010), and low-order visual factors, such as visual processing (Binder and Desai, 2011), are required. Existing neuroimaging studies demonstrated differentiated patterns of brain response in visual experts under tasks, which are modulated by their accumulated experience in a given domain. Resting-state spontaneous brain fluctuations actively encode previous learning experience. However, few studies have considered how real-world visual experience alters the level of baseline brain activity in the resting state. This study aimed to investigate how short-term real-world visual experience modulates baseline neuronal brain activity in the resting state using the amplitude of low frequency (<0.08 Hz) and a group of intern radiologists (*n* = 32). The resting-state fMRI data and the behavioral data regarding their level of visual expertise in radiology and face recognition were collected before and after 1 month of training in the X-ray department. A novel machine learning analytical method, i.e., recursive feature elimination SVM embedded in LOOCV, was used to identify subtle changes in the level of baseline brain activity (Figure 1). With a superb classification accuracy of 86.7% (Figure 3A), the results demonstrated that the left posterior cingulate cortex (CG_L_7_4), the right anterior cingulate cortex (CG_R_7_2), the left superior frontal gyrus (SFG_L_7_2), the bilateral precentral gyrus (PrG_L_6_4 and PrG_R_6_4), the bilateral superior parietal lobule (SPL_R_5_4, SPL_R_5_1, and SPL_L_5_4), the bilateral precuneus (PCun_R_4_4 and PCun_L_4_3) showed highest discriminative power after short-term visual learning (Figure 4 and Table 2). To the best of our knowledge, this study is the first to investigate the baseline neurodynamic alterations in response to real-world visual experience using longitudinal experimental design. Our findings may help develop new insights into the neural mechanism of visual experts and provide new ideas for the cultivation of visual experts.

### Increased Level of Activity in Brain Regions Supporting Working Memory

Working memory (WM) supports the online maintenance and manipulation of information without external stimulation (Baddeley, 1987). The capacity of WM serves as a reliable predictor for the performance of visual experts (Sohn and Doane, 2004). In this study, after training, the radiology interns had increased ALFF in the anterior cingulate gyrus, the posterior cingulate gyrus, and the superior frontal gyrus (Figure 4 and Table 2). Jonides (2004) reported deactivation in the anterior cingulate gyrus, which supported increased WM load under task condition. Duan et al. (2012) found that the activation of posterior cingulate gyrus was enhanced in professional chess players in the game, which was related to enhanced requirement in the WM. Teresa et al. (2018) found increased activation in the superior frontal gyrus under the visual tasks, which required online monitoring and manipulation of task-related information. In sum, all these regions, i.e., the anterior cingulate gyrus, the posterior cingulate gyrus, and the superior frontal gyrus, are closely related to the WM process. The increased level of baseline brain activity in these regions might reflect tuning with training, which in turn decreases the need for executive control in the maintenance of task-relevant information. We propose that these alterations during expertise acquisition might support more automated encoding and maintenance of objects in their expert domain, indicating a more efficient mechanism subserving visual expertise.

### Decreased Level of Activity in Brain Regions Underlying Memory Extraction

In our study, compared with the pre-training condition, the radiology interns had decreased level of ALFF in the bilateral precuneus (Figure 4 and Table 2). Visual recognition intensively depends on the retrieval of conceptual knowledge (Binder and Desai, 2011). The difference in memory extraction predicts the performance difference between visual experts and novices (Binder and Desai, 2011). Assaf et al. (2013) reported the involvement of the right precuneus in memory extraction using the visual expertise model of car experts. While in the resting-state study, Duan et al. reported the reduction of default mode network activity, including left precuneus in the professional chess players, which is closely related to episodic memory extraction. In this study, the bilateral precuneus explicated decreased level of activity after short-term visual training. Given the fact that the resting-state brain activity is involved in the coding of expected sensory stimuli (Jin et al., 2017), we propose that the tuning in these regions is likely to reflect the optimal internal coping mechanism that supports the redistribution of cognitive resources into more demanding brain process (Fox et al., 2005).

### Decrement in the Level of Activity in Brain Regions Underlying Attention Control

Visual attention is a critical component in visual recognition, which facilitates subjects to focus on the target objects in a more efficient way when dealing with complex visual scenes and gives priority to the target visual objects to ensure task completion (Cohen and Lefebvre, 2005). Therefore, the difference in the brain representation underlying attention control may serve to distinguish the brain states of experts and novices (Memmert et al., 2009). In this study, the radiology interns had decreased ALFF in the superior parietal lobule after training (Figure 4 and Table 2). Reilhac et al. (2013) reported deactivation in the right superior parietal lobules, which was closely related to visual attention in radiologists. Ouellette et al. (2020) also found deactivation in the left SPL in radiologists, which was attributed to more efficient control of visual attention supported by accelerated eye-tracking data. We propose that decreased ALFF in SPL also reflects a similar trend. After visual training, the attention control is more efficient, which gives the subjects more flexibility in manipulating attentional resources, so that the resource allocated to attention before training might be allocated later to other brain regions supporting more demanding tasks.

### Enhanced Level of Activity in Brain Regions Supporting Visual Recognition

In our study, the bilateral precentral (the PrG_L_6_4 and the PrG_R_6_4) showed enhanced ALFF after short-term visual training (Figure 4 and Table 2). Activations were found in the bilateral anterior central gyrus when visual stimuli were shown to subjects (Marks et al., 2019) and the level of brain activity increased with the number of stimuli (Mechelli et al., 2014). Studies using car experts reported an increase in gray matter volumes in this region (Gilaie-Dotan et al., 2012) and an increased level of evoked brain response to expertise-related visual stimuli in this region (Bentin, 2010). We suggest that our finding also reflects similar changes, but the exact nature of the alteration remained to be elucidated.

## Limitations

Several issues should be mentioned when the findings from this study are considered. First, the sample size is not optimal. Given the longitudinal design and the COVID pandemic, the current size is the best that can be achieved. Second, given the ratio between the number of discriminative features and the number of samples, this study faced an overfitting issue, which is quite common in MRI studies using a machine learning analytical framework. But it should be noted that three steps were taken to minimize the possibility of overfitting in our study. Particularly, a region-wise feature extraction strategy was used, which decreased the number of features from tens of thousands to 246. Then, a two-step feature selection was conducted, which decrease the number of features from 246 to 25. At last, an RFE-SVM analytical framework was employed to cut off the number of features to an optimal level, resulting in 10 features, i.e., 10 brain regions. Taken together, we do recommend further studies to repeat the current findings using larger samples. Third, for the behavioral tasks, only visual tasks were used. Tasks for WM, visual attention, and memory should be taken into consideration in future studies.

## Conclusion

Our results suggest that real-world visual experience alters the resting-state brain representation in multidimensional neurobehavioral components, which are closely interrelated with high-order cognitive and low-order visual factors, i.e., attention control, WM, memory, and visual processing. We propose that our findings are likely to help foster new insights into the neural mechanisms of visual expertise.

## Data Availability Statement

The raw data supporting the conclusions of this article will be made available by the authors, without undue reservation.

## Ethics Statement

The studies involving human participants were reviewed and approved by the Ethics Committee of the First Affiliated Hospital of Xi’an Jiaotong University. The patients/participants provided their written informed consent to participate in this study.

## Author Contributions

GS, CJ, and MD: conception and study design. HW, GS, and MD: data collection and acquisition. XZ and ZZ: statistical analysis. JS and GS: interpretation of results. JS, JW, and MD: drafting the manuscript work and revising it critically for important intellectual content. All authors approval of final version to be published and agreement to be accountable for the integrity and accuracy of all aspects of the work.

## Conflict of Interest

The authors declare that the research was conducted in the absence of any commercial or financial relationships that could be construed as a potential conflict of interest.

## Publisher’s Note

All claims expressed in this article are solely those of the authors and do not necessarily represent those of their affiliated organizations, or those of the publisher, the editors and the reviewers. Any product that may be evaluated in this article, or claim that may be made by its manufacturer, is not guaranteed or endorsed by the publisher.

## Footnotes

1
http://www.fil.ion.ucl.ac.uk/spm

2
http://rfmri.org/DPARSF

3
http://rest.restfmri.net

4
https://ww2.mathworks.cn/matlabcentral/fileexchange/75567-svm-boundary-libsvm?s_tid=srchtitle_libsvm_4
